# Down-regulation of long noncoding RNA HOXA11-AS nullifies the impact of microRNA-506–3p on chondrocytes proliferation and apoptosis in osteoarthritis

**DOI:** 10.1016/j.clinsp.2024.100393

**Published:** 2024-05-29

**Authors:** Ziyang Zhang, Renhao Guo, Chengfa Cai, Pengcheng Guo

**Affiliations:** a Department of Gdansk University of Physical Education and Sport, Start for Ph.D. in Sport & Fitness Science, Gdansk, Poland; b Department of Olympic Sports Training Theory, National University of Ukraine on Physical Education and Sport, Start for PhD. St. 1, Ukraine; c Department of Exercise Physiology and Biochemistry, Shandong Institute of Sport Science, Shandong, China; d Department of Key Lab of Aquatic Sports Training Monitoring and Intervention of General Administration of Sport of China, Faculty of Physical Education, Jiangxi Normal University, Jiangxi, China

**Keywords:** Osteoarthritis, Chondrocytes, Long noncoding rna hoxa11-as, Microrna-506–3p, PI3K/AKT/mTOR pathway, Proliferation

## Abstract

**Objectives:**

This study was directed towards exploring the impacts of lncRNA HOXA11-AS-mediated microRNA (miR)-506–3p on chondrocytes proliferation and apoptosis in osteoarthritis (OA).

**Methods:**

The articular cartilages were provided by OA patients who received total knee arthroplasty, and Human Chondrocyte (HC)-OA (HC—OA) was also attained. The miR-506–3p and HOXA11-AS expressions in articular cartilages from OA patients and HC—OA cells were analyzed via qPCR. After gain- and loss-of-function assays in HC—OA cells, MTT assay and flow cytometry (FC) were used for assessing cell viability and apoptosis, accordingly. The levels of PIK3CA, AKT, and mTOR as well as AKT and mTOR phosphorylation levels assessed using western blotting (WB). The targeting correlation of HOXA11-AS and miR-506–3p as well as miR-506–3p and PIK3CA was assessed through Dual-Luciferase Reporter gene Assay (DLRA).

**Result:**

The articular cartilages from OA patients and Human Chondrocyte (HC)-OA (HC—OA) cells showed increased HOXA11-AS and decreased miR-506–3p. Mechanistically, HOXA11-AS was capable of binding to miR-506–3p to increase PIK3CA, the target gene of miR-506–3p. miR-506–3p suppression facilitated HC—OA cell proliferation and reduced their apoptosis, which was nullified by further silencing HOXA11-AS or silencing PIK3CA. The down-regulation of HOXA11-AS disrupted the PI3K/AKT/mTOR pathway, which was counteracted by further miR-506–3p inhibition.

**Conclusion:**

The silencing of HOXA11-AS might block the PI3K/AKT/mTOR pathway through miR-506–3p up-regulation, thereby restricting HC—OA cell proliferation and provoking apoptosis.

## Highlights

• HOXA11-AS silencing restrained HC—OA cell proliferation and accelerated their apoptosis.
• HOXA11-AS overexpression increased PIK3CA expression through binding to miR-506–3p.
• HOXA11-AS overexpression represses PI3K/AKT/mTOR pathway through miR-506–3p down-regulation.

## Introduction

Osteoarthritis (OA) is a disorder relevant to articular cartilage degeneration and secondary hyperostosis. It has been reported that OA symptoms occur in 8.1 % of Chinese people over the age of 45 and severely affect their quality of life, which necessitates joint replacement or osteotomy.^1^ Moreover, chondrocytes proliferation and apoptosis assume essential roles in maintaining equilibrium between the synthesis and catabolism of cartilages. Inflammation, apoptosis, and death of chondrocytes directly cause cartilage hypertrophy and further induce OA, in which abnormally expressed genes are involved. In this regard, it's pretty valuable to understand the OA molecular mechanism.

As transcripts with lengths exceeding 200 nucleotides, lncRNAs are extensively associated with biological processes like cell proliferation, apoptosis, inflammation, and tumorigenesis, and also play an essential part in OA pathogenesis. Li Y et al. found that lncRNA PVT1 functioned as a microRNA (miR)−488–3p sponge to orchestrate chondrocyte apoptosis in OA, which suggested lncRNA PVT1 as a novel treatment target for OA.^2^ HOXA11-AS is a recently discovered lncRNA, which shows high expression in gastric cancer, colorectal cancer, cervical cancer, and other tumors,^3^^,^^4^ thus affecting the occurrence and development of tumors. Cao K et al. displayed that HOXA11-AS expression in osteosarcoma cells and tissues exceeded that in adjacent tissue of osteosarcoma and human osteoblast cell lines.^5^ However, there has hitherto been little research about the impact of HOXA11-AS on articular chondrocytes. This study was conducted to ascertain whether HOXA11-AS manipulated articular chondrocytes proliferation and apoptosis. miRs are a kind of small non-coding RNAs composed of around 22 nucleotides, which can bind to specific mRNA to repress the expression of post-transcriptional genes, thereby taking part in biological processes like cell proliferation, migration as well as apoptosis. miR-506–3p is a recently discovered miR. Dinesh P et al. indicated that miR-506–3p possesses a high binding affinity to NFATc1 to control the generation of osteoclasts mediated by RANKL/NFATc1 and modulate the metabolism of bone tissues.^6^ However, it is little known about its part in OA chondrocytes proliferation and apoptosis. It has been documented that lncRNAs can specifically bind to miRNAs to regulate cells. Accumulating evidence has indicated targeting relationships of miR-506–3p with the catalytic subunit alpha of phosphoinositide-3-kinase (PIK3CA)^7^^,^^8^ and HOXA11-AS^9^ that miR-506–3p might target PIK3CA, while HOXA11-AS might target miR-506–3p to sponge miR-506–3p. However, it remains enigmatic about the role of the genetic network aforementioned in chondrocyte proliferation or apoptosis during OA. Therefore, this study intended to examine the specific mechanism of HOXA11-AS on OA chondrocyte proliferation and apoptosis with the involvement of miR-506–3p, thereby providing novel evidence in theory for OA treatment.

## Materials and methods

### Patients and tissue samples

Articular cartilage was harvested from 28 OA patients (15 males and 13 females, aged 51.9 ± 12.9) who underwent total knee arthroplasty at the Gdansk University of Physical Education and Sport. The following were inclusion criteria: 1) Patients who met OA diagnosis criteria specified by the American College of Rheumatology; 2) Patients who received no other relevant treatment before admission; 3) Patients with detailed clinical data. Besides, the following were exclusion criteria: 1) Patients with rheumatic, rheumatoid, or secondary OA; 2) Patients with major diseases such as abnormal renal or liver function or cardiac insufficiency; 3) Patients with mental disorders or dysautonomia. Meanwhile, normal cartilages were provided by 16 patients (7 males and 9 females, aged 49.6 ± 10.9) without OA or rheumatoid arthritis that went through amputation. This work has gotten the approval of the medical ethics committee of the Gdansk University of Physical Education and Sport (approval number IRB-JXNU-KLAMI-GASC-2,023,015). The study followed the STROBE statement.

### Cell cultivation

Both human chondrocytes (HC, CCTCC No.: GPC0134; species: human; source: surgical specimen; purchased from China Center for Type Culture Collection) and Human Chondrocytes – Osteoarthritis (HC—OA, adult; purity specification: 1 × 10^6^/T25; Art. No.: C7001; purchased from Shanghai Yaji Biotechnology Co., Ltd.) were cultivated at 37 °C with 5 % CO_2_ in DMEM supplemented 10 % FBS and 1 % penicillin/streptomycin. Cells were trypsinized and passed 2‒3 times. Follow-up experiments were conducted when the cell confluence reached 95 %‒100 %.

### Cell transfection

When reached 95 %‒100 % confluence, cells were inoculated into 6-well plates (5 × 10^4^ cells/well). After the cell confluence got to 90 %, the medium was renewed with an FBS-free medium. Cells were randomly divided into the siNC group (transfected with siRNA-NC), si-HOXA11-AS group (transfected with HOXA11-AS-siRNA), pcDNA group (transfected with pcDNA3.1 empty vector), pcDNA-HOXA11-AS group (transfected with pcDNA3.1-HOXA11-AS), miR-NC group (transfected with miR-NC), miR-506–3p group (transfected with anti-miR-506–3p), si-HOXA11-AS + anti-miR-NC group (co-transfected with HOXA11-AS-siRNA and anti-miR-NC), si-HOXA11-AS +anti-miR-506–3p group (co-transfected with HOXA11-AS-siRNA and anti-miR-506–3p). Strictly as per the protocols of Lipofectamine 2000 (Invitrogen, Carlsbad, California, USA), miR-506–3p mimic and inhibitor, si-HOXA11-AS, pc-HOXA11-AS, and their respective Negative Controls (NCs) were transfected into human HC—OA cell lines. Cells were spread with 40×50×10^4^ cells/well 24 h before transfection. The fresh medium was changed about 1 h before transfection. Plasmid DNA (4 ug) and DMEM solution (250 uL) were added to a centrifuge tube, mixed well, and placed at room temperature for about 5 min. A sterile centrifuge tube was taken and added with 250 UL of DMEM medium and then added with 5‒10 UL of LIP2000 transfection reagent to mix and let stand at room temperature for 5 min. Excessive incubation should be avoided to prevent compromising cell viability. The mixed diluted DNA and the mixed diluted Lip2000 were mixed well and incubated at room temperature for 20 min. The solution might become turbid during the mixing process, yet typically, it did not affect the transfection efficiency. The incubated complex was added evenly to the culture plate, gently lifted, and shaken well. After that, it was incubated again for 6‒12 h at 37 °C in a 5 % CO_2_ incubation environment, and the fresh medium was replaced; 0.5d after transfection, cells were cultivated in a renewed medium for 2d for the subsequent experiments. qPCR was implemented to detect miR-506–3p and HOXA11-AS expressions to verify transfection efficiency. The miR-506–3p mimic, miR-506–3p inhibitor, sh-HOXA11-AS, pc-HOXA11-AS, and their respective negative controls were purchased from Suzhou Genepharma Co., Ltd.

### qPCR

After being isolated from cells and tissues via TRIzol reagent, RNA concentration and purity were measured via a low-volume spectrometer, and pure RNA had an A260 nm/A280 nm ratio of 1.8‒2.0. The extracted RNA was reversely transcribed to cDNA through a TaqMan MicroRNA qPCR kit (GeneCopoeia, Rockville, MD, USA). The cDNA served as a template amplified with the SYBR®Green to assess HOXA11-AS expression. iQTM5 Multicolor Real-Time PCR Detection System was utilized for analyzing miR-506–3p and PIK3CA expressions. The relative target gene expressions were obtained via the formula: 2^−△△Ct^ with GAPDH as HOXA11-AS and PIK3CA normalizer, and U6 as miR-506–3p normalizer.

### MTT assay

A single-cell suspension (3 × 10^4^ cells/mL) was obtained using cells at the logarithmic growth phase and inoculated in a 96-well plate for corresponding treatment. Each group had three wells and cells were cultivated for 3d at 37 °C containing 5 % CO_2_. Serum-free medium encompassing MTT solution (5 mg/mL, 10 µL) was supplemented at the 24th, 48th, and 72th hour, respectively. Following 4-hour incubation at 37 °C, the supernatant was removed from the culture well, and DMSO (150 μL) was then added. A microplate reader was utilized for demonstrating OD_450 nm_ (Optical Density).

### Flow cytometry (FC)

A single-cell suspension (2 × 10^5^ cells/mL) was obtained, and 1 mL suspension was centrifugated for 5 min at 1000 r/min. The supernatant was deserted, and cells were rinsed three times using phosphate buffer saline. Then, the buffer solution was added at the ratio of 1:1 to resuspend the cells, which were supplemented Annexin V-fluoresceinisothiocyanate (5 μL) and propidium iodide (10 μL), respectively, and reacted at rt for 10 min away from lights. The apoptosis rate was detected through a flow cytometer (Beckman Coulter Company, Brea, CA, USA). The sum of the early and late apoptosis rates was considered the total apoptosis rate (%).

### Western blotting (WB)

After being extracted from cells, the protein was subjected to concentration measurement through the BCA method. Next, the protein was degraded and loaded, isolated through SDS-PAGE, and electrically added to PVDF membranes, which were subsequently blocked-in buffer encompassing 5 % skimmed milk powder for 60 min, followed by overnight probing with primary rabbit anti-human antibodies to Bcl-2, Bax, Caspase-3, PIK3CA, and GAPDH (manufacturer, cat., 1:500). After washed three times containing Tris-buffered saline added Tween 20, the membrane was subjected to incubation using goat anti-rabbit Immunoglobulin G secondary antibody (manufacturer, cat., 1:2000) conjugated to horseradish peroxidase at 37 °C for 60 min. The protein bands were observed through electrogenerated chemiluminescence, followed by OD analysis of the bands. The relative protein expression was displayed as the grayscale values of target bands versus those of the internal control band.

### Dual-luciferase reporter gene assay (DLRA)

The bioinformatics online predictor software Starbase (http://starbase.sysu.edu.cn) was utilized for predicting the binding sites between HOXA11-AS and miR-506–3p. The plasmids containing the HOXA11-AS 3′Untranslated Region (UTR), Wild Type (WT) fragment, and the HOXA11-AS 3′UTR Mutant (MUT) fragment were designed and synthesized by GenePharma (Shanghai, Chian), and cloned into psiCHECK2. Luciferase reporter vectors HOXA11-AS 3′UTR-WT and HOXA11-AS 3′UTR-MUT were constructed and co-transfected into 293T with miR-506–3p mimic and miR-NC, respectively. Cells were attained 1d after transfection. DLRA kit was applied to assess luciferase activity.

### Data analysis

Data were analyzed through SPSS 22.0 and measurement results were shown as mean ± SD. *t*-test, one-way ANOVA and Least Significant Difference test were utilized for analyzing the comparisons between two groups, among multiple groups and pairwise comparisons, respectively; *p* < 0.05 meant a considerable distinction in statistics.

## Results

### HOXA11-AS showed high level and miR-506–3p showed low level in OA articular cartilages

**Fig. 1:**
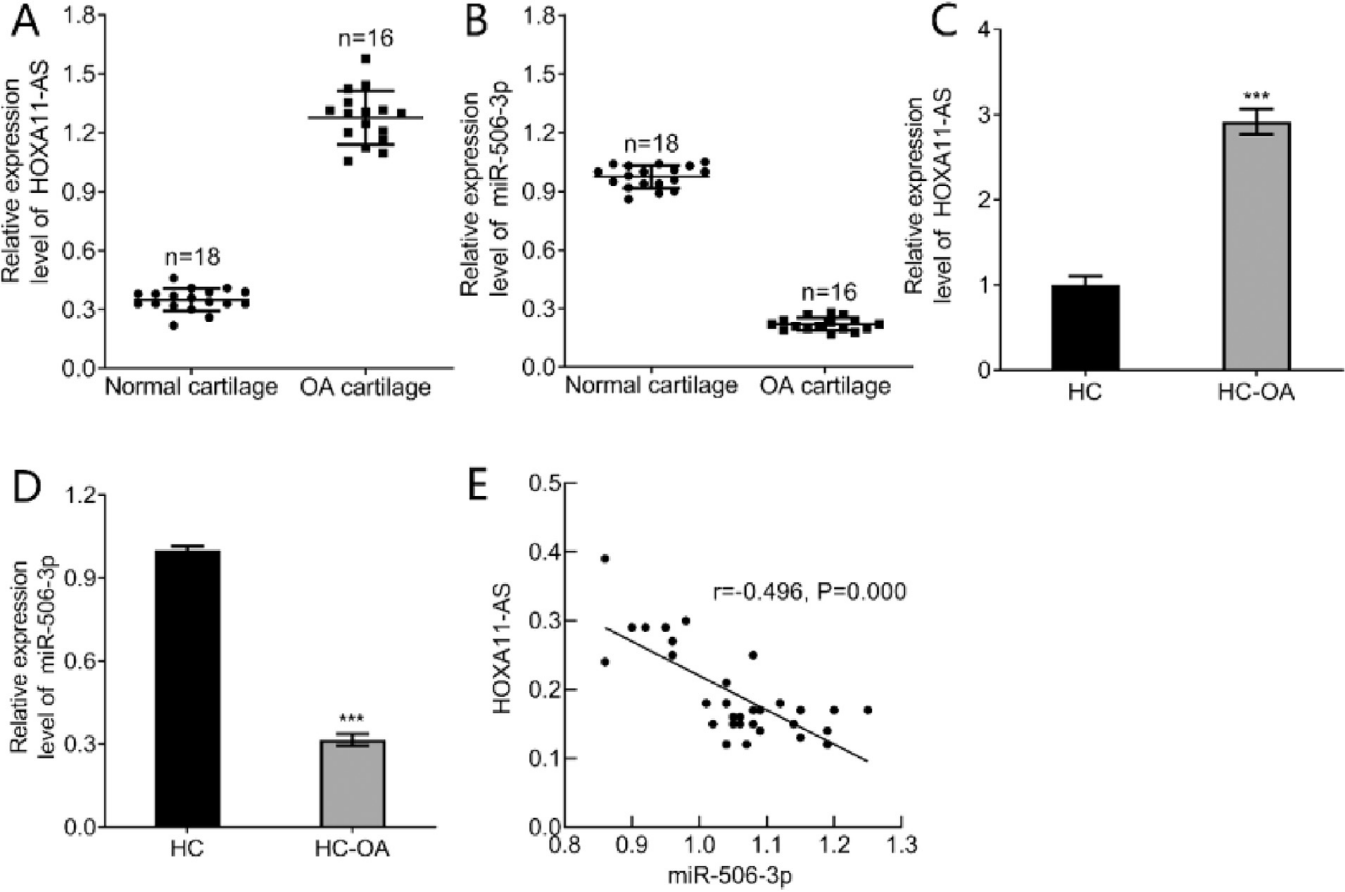
HOXA11-AS increase and miR-506–3p decrease are observed in OA articular cartilages. (A) qPCR detection of the HOXA11-AS expression in OA patients’ articular cartilages. (B) The miR-506–3p expression in OA patients’ articular cartilages measured using qPCR. (C) The HOXA11-AS expression in HC—OA and HC cells assessed by qPCR. (D) qPCR to evaluate the miR-506–3p expression in HC—OA and HC cells. (E) Correlation analysis between miR-506–3p and HOXA11-A expression in OA patients’ articular cartilages. *** *p* < 0.001 vs. normal cartilages or HC cells.

Initially, qPCR was applied to assess the HOXA11-AS and miR-506–3p expressions in OA patients’ articular cartilages to figure out their parts in OA, which showed that HOXA11-AS expression showed significantly higher expression but miR-506–3p showed obviously poorer expression in OA articular cartilages relative to in healthy cartilages (*p* < 0.001; Fig. 1A‒B). The authors further measured the miR-506–3p and HOXA11-AS expressions in HC—OA, which displayed that HOXA11-AS showed remarkably upregulated expression whereas miR-506–3p showed considerably reduced expression in HC—OA in contrast to HC (*p* < 0.001; Fig. 1C‒D). Correlation analysis revealed the inverse relationship of miR-506–3p and HOXA11-AS expressions in OA patients’ articular cartilages (*r* = −0.496, *p* < 0.001; Fig. 1E). These findings displayed HOXA11-AS increase and miR-506–3p decrease in OA articular cartilages.

### HOXA11-AS down-regulation suppressed HC-OA cell proliferation and induced apoptosis

**Fig. 2:**
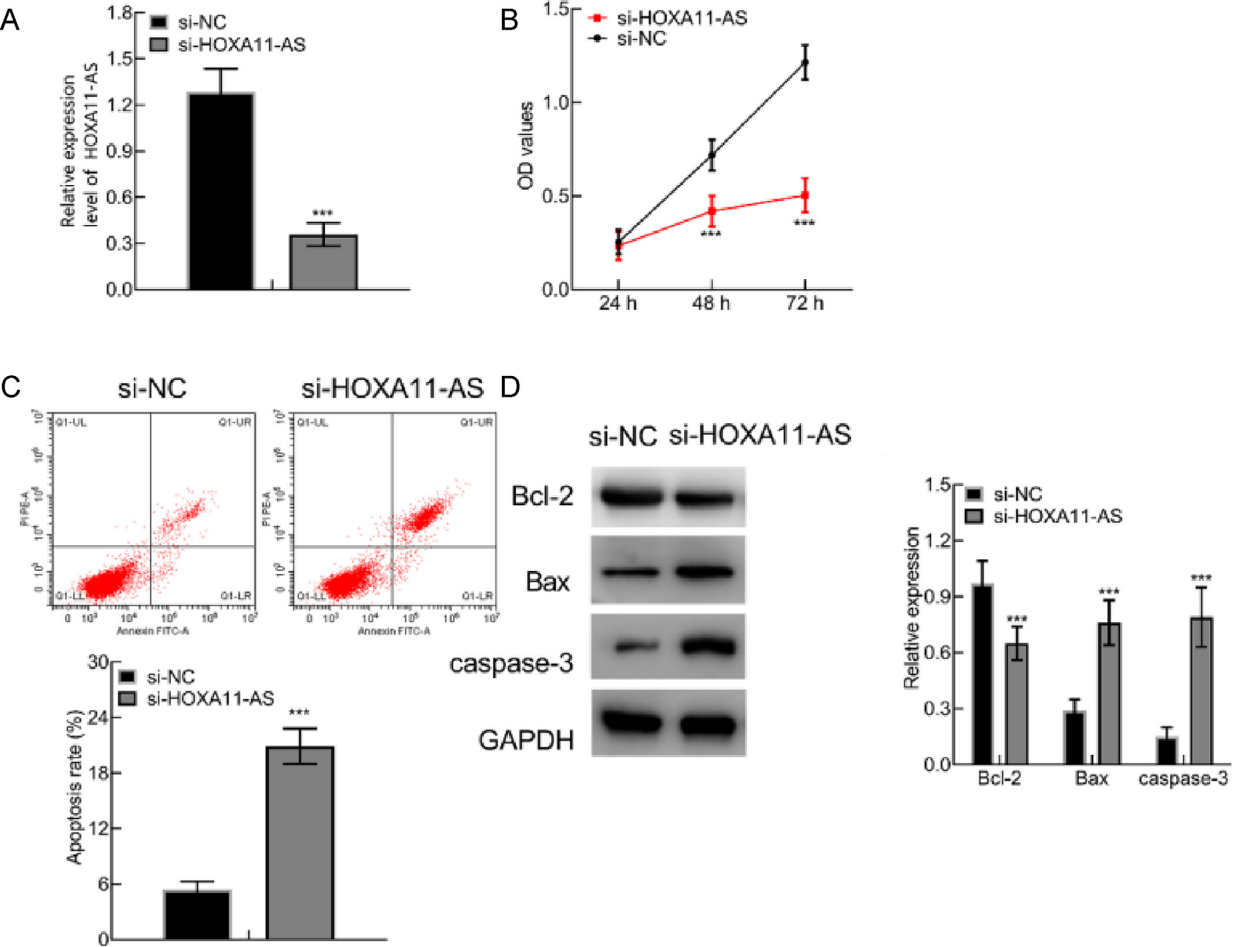
HOXA11-AS silencing causes repressed HC—OA cell proliferation and enhanced apoptosis. (A) HOXA11-AS expression in HC—OA cells after HOXA11-AS silencing measured using qPCR. (B) HC—OA cell viability after HOXA11-AS silencing detected using MTT assay. (C) HC—OA cell apoptosis rate after HOXA11-AS silencing measured using FC. (D) WB of the expression of apoptosis-relevant proteins in HC—OA cells after HOXA11-AS silencing. *** *p* < 0.001 vs. the si-NC group.

To further ascertain the influence of HOXA11-AS, siRNAs were transfected into HC—OA cells to reduce HOXA11-AS expression. In contrast to si-NC group, HOXA11-AS level in si-HOXA11-AS group was potently reduced (*p* < 0.001; Fig. 2A). MTT assay displayed that relative to si-NC group, the HC—OA cell viability in si-HOXA11-AS group diminished conspicuously after 48- and 72-hour cell culture (*p* < 0.001; Fig. 2B). FC indicated that the HC—OA cell apoptosis rate in si-HOXA11-AS group was substantially higher over that in si-NC group (*p* < 0.001; Fig. 2C). WB documented that anti-apoptotic protein Bcl-2 expression was lowered, whereas si-HOXA11-AS group exhibited elevated Caspase-3 and Bax expressions than si-NC group (*p* < 0.001, Fig. 2D). Conclusively, HOXA11-AS silencing restrained HC—OA cell proliferation and accelerated their apoptosis.

### HOXA11-AS was capable of binding to miR-506–3p to increase PIK3CA

**Fig. 3:**
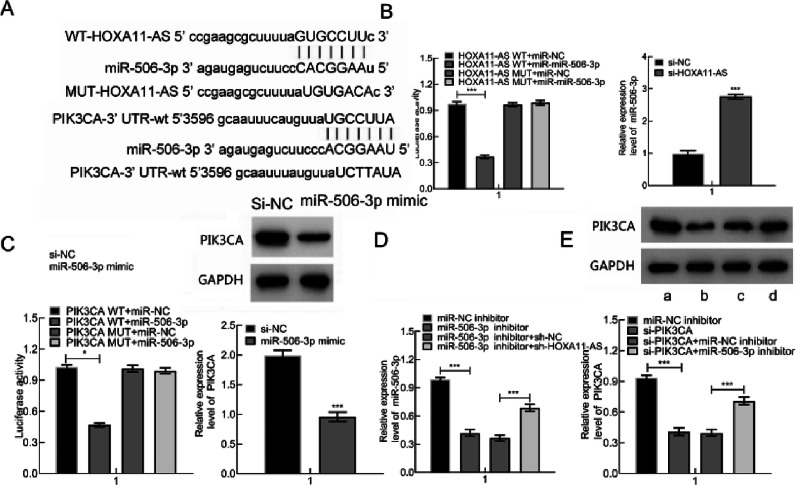
HOXA11-AS was capable of binding to miR-506–3p that inversely targets PIK3CA. (A) The predicted binding sites between HOXA11-AS and miR-506–3p as well as miR-506–3p and PIK3CA. (B) The binding correlation evaluated by DLRA and miR-506–3p expression in cells after HOXA11-AS silencing measured by qPCR. (C) DLRA to evaluate the targeting correlation of PIK3CA and miR-506–3p and PIK3CA expression in cells after miR-506–3p mimic transfection. (D) miR-506–3p level following miR-506–3p mimic and/or si-HOXA11-AS transfection assessed using qPCR. (E) PIK3CA expression in cells following si-PIK3CA and/or miR-506–3p inhibitor transfection. a: miR-NC inhibitor; b: si-PIK3CA; c: si-PIK3CA + miR-NC inhibitor; d: si-PIK3CA + miR-506–3p inhibitor. * *p* < 0.05.

It's been reported that OA occurrence and progression are associated with miR-506–3p and HOXA11-AS.^5^^,^^6^ Therefore, the authors further probed the relationships among miR-506–3p, HOXA11-AS and downstream gene PIK3CA. The target genes were predicted through the Starbase website, which displayed that miR-506–3p was capable of binding to HOXA11-AS 3′UTR and PIK3CA 3′UTR (Fig. 3A).

Therefore, the authors predicted that HOXA11-AS might orchestrate PIK3CA through binding to miR-506–3p. DLRA illustrated that miR-506–3p mimic was capable of decreasing HOXA11-AS-WT luciferase activity while not of HOXA11-AS-MUT. Subsequent to si-HOXA11-AS transfection into cells, miR-506–3p expression was clearly augmented (Fig. 3B). Also, miR-506–3p mimic was capable of triggering the decline in PIK3CA-WT luciferase activity while not of PIK3CA-MUT. Besides, miR-506–3p mimic transfection contributed to remarkable reduction in PIK3CA protein expression in cells (Fig. 3C). Besides, down-regulate miR-506–3p caused by miR-506–3p inhibitor was nullified by further si-HOXA11-AS treatment (Fig. 3D), and down-regulated PIK3CA induced by si-PIK3CA was negated by further miR-506–3p inhibitor treatment (Fig. 3E). In summary, HOXA11-AS overexpression increased PIK3CA expression through binding to miR-506–3p.

### HOXA11-AS silencing suppressed HC-OA cell proliferation and facilitated their apoptosis through reducing PIK3CA expression via miR-506–3p up-regulation

**Fig. 4:**
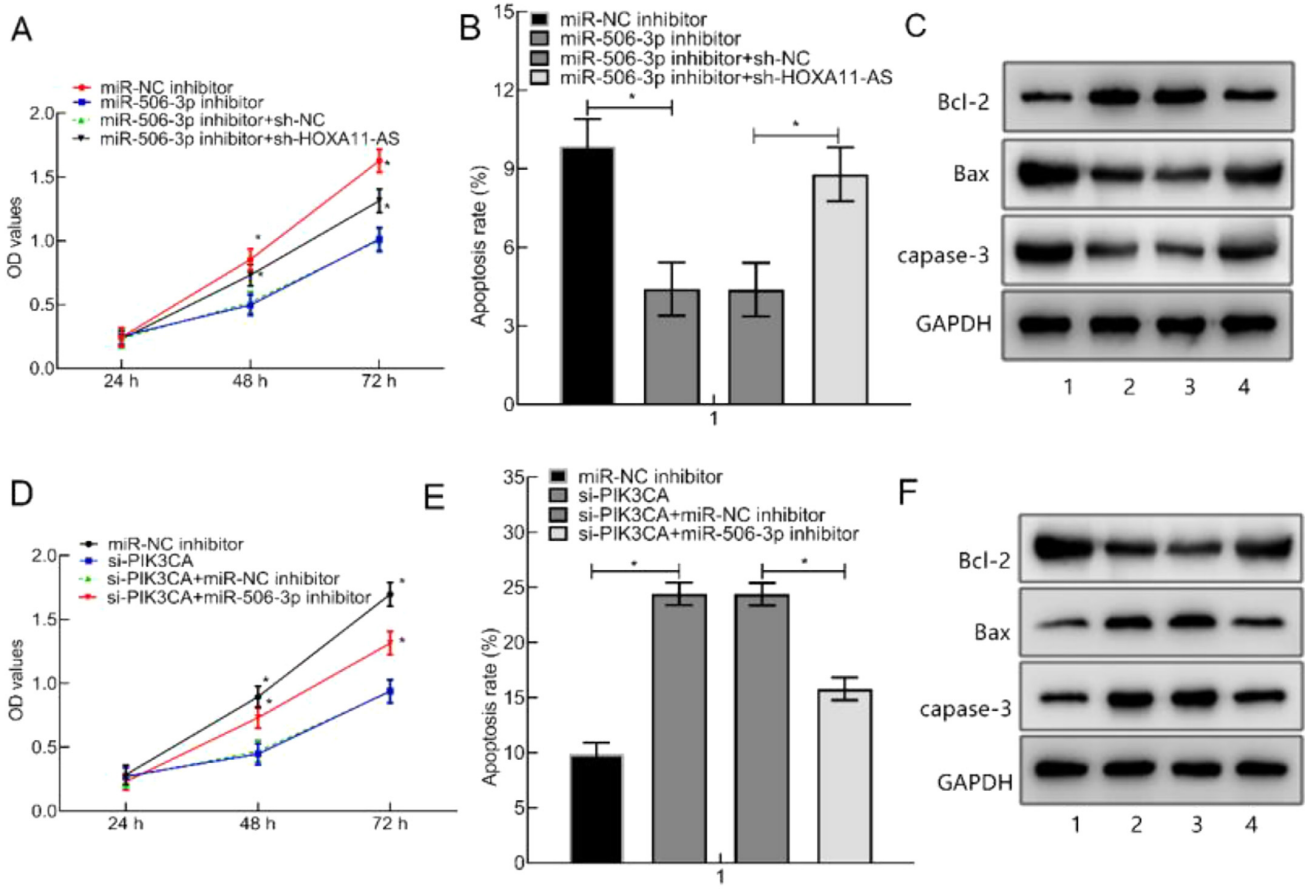
HOXA11-AS down-regulation inhibits HC—OA cell proliferation and induces apoptosis through orchestrating miR-506–3p/PIK3CA axis. (A) MTT assay to detect HC—OA cell viability after si-HOXA11-AS and miR-506–3p inhibitor transfection. (B) FC of HC—OA cell apoptosis rate after transfection. (C) WB of the expression of apoptosis-relevant proteins in HC—OA cells after transfection. (D) HC—OA cell viability after transfection measured by MTT assay. (E) FC to assess HC—OA cell apoptosis rate after transfection. (F) The expression of apoptosis-relevant proteins in HC—OA cells after transfection evaluated by WB. *p < 0.05.

Then, the focus shifted to the impacts of HOXA11-AS/miR-506–3p/PIK3CA axis on OA by transfecting HC—OA cells with miR-506–3p inhibitor in combination with si-PIK3CA or si-HOXA11-AS. The results manifested an elevation in the viability and a decline in the apoptosis of HC—OA cells after miR-506–3p inhibitor treatment, accompanied by reduced Caspase-3 and Bax and increased Bcl-2, which was counterweighed by further si-HOXA11-AS treatment (Fig. 4A‒C). When si-PIK3CA was transfected into HC—OA cells alone or in combination with miR-NC inhibitor, cell viability was obviously diminished, and cell apoptosis rate was augmented evidently, accompanied by enhanced Caspase-3 and Bax and decreased Bcl-2. These trends were annulled by further miR-506–3p inhibitor treatment (Fig. 4D‒F). Collectively, HOXA11-AS silencing restricted HC—OA cell proliferation and facilitated apoptosis through miR-506–3p/PIK3CA axis.

### HOXA11-AS silencing suppressed PI3K/AKT/mTOR pathway through increasing miR-506–3p

**Fig. 5:**
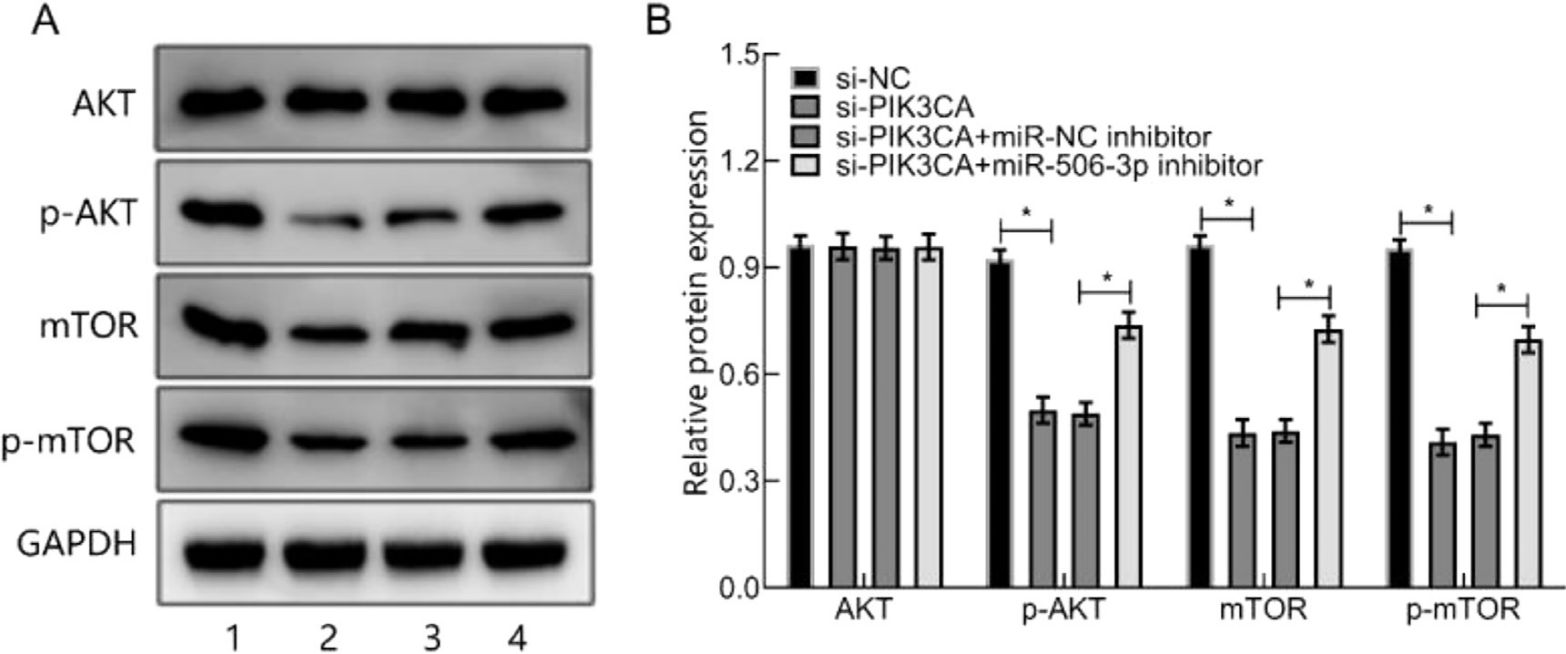
HOXA11-AS down-regulation inactivates PI3K/AKT/mTOR pathway through miR-506–3p. (A) PI3K/AKT/mTOR western blot band images. B: Bar graphs of PI3K/AKT/mTOR pathway-related protein expression. 1. The si-NC group; 2. The si-HOXA11-AS group; 3. The si-HOXA11-AS + miR-NC inhibitor group; 4. si-HOXA11-AS + miR-506–3p inhibitor. * *p* < 0.05.

Prior research has reported that the PI3K/AKT/mTOR pathway is tightly linked to OA pathogenesis.^10^ To deeply study the regulation of HOXA11-AS on miR-506–3p downstream pathway, WB was utilized for determining the level of PI3K/AKT/mTOR pathway-relevant proteins in miR-506–3p downstream. The results depicted that HOXA11-AS silencing diminished the level of PIK3CA and mTOR proteins as well as AKT and mTOR phosphorylation levels, but did not change AKT expression, which was offset by further miR-506–3p inhibitor treatment (Fig. 5). These findings displayed that HOXA11-AS downregulation blocked PI3K/AKT/mTOR pathway by increasing miR-506–3p expression.

## Discussion

The OA pathogenesis is linked to the interaction of multiple factors,^11^ among which lncRNAs are critical. It has been manifested that lncRNAs, such as GAS5, PCGEM1, lncRNA CIR, and HOTAIR, can effectively promote OA. The data obtained from the present work elucidated that HOXA11-AS expression in OA cartilage was upregulated, which facilitated chondrocytes proliferation and disease development by activating the PI3K/AKT/mTOR pathway via miR-506–3p downregulation.

Kun Cao et al. displayed that HOXA11-AS might manipulate osteosarcoma occurrence and development. Chen JH et al. indicated that HOXA11-AS overexpression was capable of suppressing cell proliferation and facilitating apoptosis,^12^ which contribute to fracture healing. However, its impact on articular chondrocytes remains poorly identified. the present study elaborated that HOXA11-AS in OA cartilages and chondrocytes was increased and that HOXA11-AS down-regulation suppressed HC—OA cell proliferation and accelerated their apoptosis. Meanwhile, the Bcl-2 level was decreased and the Caspase-3 and Bax expressions were increased, suggesting that HOXA11-AS down-regulation was capable of inhibiting OA chondrocytes proliferation and induce apoptosis.

Previous research has observed that lncRNAs can bind to specific miRs, leading to diseases.^13^^,^^14^ According to Wang G et al.^15^ lncRNA UCA1 could enhance MMP-13 level through repressing miR-204–5p in chondrocytes. Chen K et al. reported that lncRNA MEG3 could restrain the chondro-extracellular matrix degradation in OA by acting on the miR-93/TGFBR2 axis.^16^ The authors observed that miR-506–3p expression in OA cartilages and chondrocytes was reduced and inversely linked to HOXA11-AS expression. DLRA demonstrated that HOXA11-AS was able to target miR-506–3p. Additionally, miR-506–3p overexpression could restrict OA chondrocytes proliferation and accelerate apoptosis, contrary to the effect of HOXA11-AS.

PI3K/AKT/mTOR pathway is a key pathway in OA occurrence and progression,^17^, ^18^, ^19^, ^20^, ^21^ whose activation promotes the proliferation and cell cycle progression of chondrocytes.^22^^,^^23^ The study discovered that miR-506–3p was able to target PIK3CA and PIK3CA silencing also repressed OA chondrocytes proliferation and facilitated their apoptosis.

Besides, the authors also uncovered that miR-506–3p down-regulation could counteract the influence of HOXA11-AS silencing on OA chondrocytes. The si-PIK3CA transfection could suppress OA chondrocytes proliferation and promote apoptosis, which was negated by inhibiting miR-506–3p. Thus, the authors believed that down-regulated HOXA11-AS could enhance miR-506–3p expression to repress PIK3CA. To further understand whether HOXA11-AS modulates the PI3K/AKT/mTOR pathway, WB was utilized for assessing the levels of pathway-relevant proteins. The authors unraveled that HOXA11-AS silencing led to the repression of PIK3CA and mTOR expressions as well as AKT and mTOR phosphorylation levels. The miR-506–3p inhibitor restored the diminished expression of proteins induced by si-HOXA11-AS. These results suggest that HOXA11-AS modifies the phosphorylation of AKT, an upstream protein of mTOR, by mediating PIK3CA. However, the network of other interactions among miRs and their target genes is more complex and is yet to be elucidated.

In conclusion, HOXA11-AS overexpression represses the PI3K/AKT/mTOR pathway through miR-506–3p down-regulation, which accelerates chondrocyte proliferation and then promotes the development of OA.

## CRediT authorship contribution statement

**Ziyang Zhang:** Data curation, Investigation, Resources, Writing – original draft, Writing – review & editing. **Renhao Guo:** Formal analysis, Methodology. **Chengfa Cai:** Data curation, Project administration. **Pengcheng Guo:** Conceptualization, Methodology, Software, Writing – original draft, Writing – review & editing.

## Declaration of competing interest

The authors declare no conflicts of interest.
